# Sustainable Leadership Promotes Employees Taking Charge in Green Production: A Resource Investment Perspective

**DOI:** 10.3390/bs15121691

**Published:** 2025-12-06

**Authors:** Zengguang Fan, Zhongming Wang, Honghao Hu, Jinjin He, Yuechao Du

**Affiliations:** 1 Department of Psychology and Behavioral Sciences, Zhejiang University, Hangzhou 310058, China; psyfanzg@163.com; 2 Global Entrepreneurship Research Center, Zhejiang University, Hangzhou 310058, China; zmwzju@foxmail.com (Z.W.); hejinjin_hjj@163.com (J.H.); 3 School of Management, Zhejiang University, Hangzhou 310058, China; 4 Postdoctoral Research Station, China Orient Asset Management Co., Ltd., Beijing 100033, China; ycdu@zju.edu.cn

**Keywords:** sustainable leadership, taking charge, employee resilience, colleague support, green production

## Abstract

Sustainable performance in green production hinges on collective employee engagement. While prior research has largely focused on the influence of sustainable leadership in fostering employee role behaviors and team relationship-oriented behaviors, this study delves into the critical role of taking charge behavior, which is related to the organization’s additional performance growth and long-term development. This study, grounded in the Conservation of Resources theory, explores how sustainable leadership encourages taking charge behavior through employee resilience as a mediator and colleague support as a moderator. Using the longitudinal method, data from 386 paired responses were collected from corporate employees across two time periods. Hypotheses were tested using hierarchical regression analysis, supplemented by path analysis to explore mediating and moderating effects. The findings indicate that sustainable leadership can enhance taking charge behavior by strengthening employee resilience, and in environments with robust colleague support, the impact of sustainable leadership on improving employee resilience is magnified, resulting in a more effective promotion of taking charge. This study contributes both theoretically and practically to the field of sustainable leadership.

## 1. Introduction

Organizations worldwide are increasingly adopting green production initiatives (C. Wang & Zhang, 2020). Leadership plays a pivotal role in driving this transformation by integrating environmental and social objectives into strategic decision-making (Avery & Bergsteiner, 2011). Among leadership approaches, sustainable leadership has gained prominence as a future-oriented style grounded in the triple bottom line principle, balancing organizational success with social equity and environmental stewardship (McCann & Sweet, 2013; Zada et al., 2024). By emphasizing long-term viability over short-term gains, sustainable leadership catalyzes sustainable organizational change (Hu et al., 2023), attracting increasing scholarly interest.

Prior research on sustainable leadership has largely focused on three key domains: (1) its impact on performance outcomes, such as financial results (Suriyankietkaew & Avery, 2016), project success (Zada et al., 2024), and sustainability metrics (Iqbal et al., 2020); (2) its role in promoting innovation, especially in environmental and resource-constrained contexts (Hu et al., 2023; Ur Rehman et al., 2024); and (3) its influence on employee development, including psychological resilience and well-being (Iqbal & Piwowar-Sulej, 2022; Choi, 2021). More recently, research has begun to highlight sustainable leadership’s role in fostering behaviors vital to organizational transformation, such as green innovation and organizational citizenship behaviors (Mandal & Pal, 2025; Choi, 2021; Javed et al., 2021). Despite these advancements, one critical but underexplored behavior is taking charge, defined as voluntary, constructive efforts to initiate change in work methods (Morrison & Phelps, 1999). Unlike innovation or organizational citizenship behavior (OCB), taking charge is a high-risk, discretionary behavior that directly challenges the status quo to improve organizational functioning (Tang et al., 2025; Moon et al., 2008). This bottom-up change complements leadership efforts by empowering employees to address inefficiencies and outdated practices, thereby driving sustainable organizational development (Lozano, 2015). Despite its significance, the relationship between sustainable leadership and taking charge behavior remains unexplored. Therefore, this research aims to explore the impact of sustainable leadership on employees’ taking charge behavior in the context of green production and uncover the underlying mechanisms of this relationship.

Existing theoretical frameworks explaining the influence of sustainable leadership on employee behaviors typically rely on Social Learning Theory, emphasizing observational learning from leaders (Javed et al., 2021), or Social Exchange Theory, underscoring reciprocal exchanges driven by perceived support (Choi, 2021). Although these theories provide valuable insights, they inadequately address motivational processes central to sustainable leadership—such as job security, participative decision-making, and inclusive organizational climates—that inherently encourage employees to proactively initiate actions aimed at personal and organizational development rather than merely responding to external demands (Avery & Bergsteiner, 2011; Piwowar-Sulej & Iqbal, 2023). Moreover, existing theories fall short in explaining why employees engage in inherently risky, discretionary proactive behaviors. To bridge this gap, the current study adopts the Conservation of Resources (COR) theory (Hobfoll et al., 2018), conceptualizing employee motivation as being driven by the acquisition, accumulation, and strategic investment of valuable resources.

The COR theory posits that individuals are inherently driven to acquire and invest valuable resources to safeguard future resource needs and achieve sustained growth (Hobfoll et al., 2018). Building upon this premise, we argue that sustainable leadership enhances employees’ access to essential resources—including leaders’ support, job security, and developmental feedback—thus stimulating intrinsic motivation to engage in proactive resource investment behaviors, notably taking charge (Braun & Nieberle, 2017; Xu et al., 2023). This process is dynamic, involving an initial phase of acquiring external resources followed by the strategic investment of these amassed reserves. Within this framework, internal resources play a pivotal role in bridging these two stages. Furthermore, given that taking charge involves inherent risks such as potential rejection or failure, employees require sufficient psychological resilience—a crucial psychological resource enabling effective management of stress and uncertainty—to successfully navigate and overcome these barriers (Avey et al., 2009; Hobfoll et al., 2018). Consequently, this research proposes employee resilience as a pivotal mediating mechanism through which sustainable leadership positively influences taking charge behavior.

However, the capacity for employees to acquire resources from sustainable leaders is not limitless and is significantly shaped by contextual factors (Hobfoll et al., 2018). Colleague support, in particular, emerges as a crucial contextual influence (Maria Stock et al., 2017). Contemporary workplaces often exhibit both cooperative and competitive interpersonal dynamics (Eisenkraft et al., 2017), implying that colleagues can either facilitate or impede each other’s resource acquisition efforts, especially under conditions of resource scarcity. Therefore, this study proposes colleague support as a critical boundary condition moderating the relationship between sustainable leadership and taking charge behaviors. Leveraging the COR theory’s Resource Caravan Passageways principle (Hobfoll et al., 2018)—which emphasizes that resources accumulate progressively in interaction with the environmental context, notably colleague interactions (S. Chen et al., 2015)—this research examines how colleague support operates as a catalyst, enhancing employees’ willingness and ability to access resources provided by sustainable leaders, thereby amplifying the motivational effects of sustainable leadership on taking charge behavior.

Despite these theoretical insights, several key scientific problems remain insufficiently addressed. It is still unclear whether sustainable leadership can motivate employees to engage in taking charge behavior—a high-risk proactive behavior essential for green production. Existing theories also provide limited explanations for the internal psychological mechanism through which sustainable leadership fosters such discretionary, resource-intensive behavior. Additionally, little is known about the contextual conditions under which sustainable leadership is most effective in promoting proactive resource investment. To address these issues, this study investigates the effect of sustainable leadership on taking charge behavior, the mediating role of resilience, and the moderating role of colleague support based on the Conservation of Resources theory.

In summary, this study develops and empirically tests a comprehensive theoretical model based on the COR theory, aiming to clarify both the mediating mechanisms and boundary conditions underlying the influence of sustainable leadership on employees’ taking charge behavior. By doing so, this research contributes significantly to the existing literature on sustainable leadership and green production practices in three key ways. First, it expands the scope of sustainable leadership effectiveness by extending its influence beyond conventional role-based and team relationship-oriented behaviors to include proactive behaviors critical for achieving sustainable organizational transformation. Second, this study unveils the mechanisms through which sustainable leadership fosters proactive yet risky behaviors. By introducing psychological resilience as a mediator grounded in the COR theory, the research elucidates how sustainable leadership facilitates proactive behaviors by enhancing employees’ psychological resources, thereby stimulating intrinsic motivational processes. This contribution further broadens the theoretical application of the COR theory within sustainability contexts. Third, the study reveals the dynamic role of contextual boundary conditions. Responding to scholarly calls for identifying additional boundary conditions influencing sustainable leadership effectiveness, the study highlights colleague support as a critical contextual moderator. Specifically, it clarifies the conditions under which sustainable leadership most effectively promotes employee resource acquisition and proactive motivational processes, thus enriching the understanding of sustainable leadership dynamics from a resource-based perspective.

## 2. Theory and Hypotheses

### 2.1. Sustainable Leadership and Taking Charge Behavior

Over the past decade, sustainable leadership has emerged as a transformative leadership paradigm, gaining increasing scholarly and practical attention due to its dynamic and future-oriented characteristics. Rooted in the triple bottom line principle, sustainable leadership encompasses values, principles, and organizational practices that collectively aim to balance economic viability, social equity, and environmental responsibility (Suriyankietkaew & Avery, 2016; McCann & Sweet, 2013).

Sustainable leaders are distinguished by their long-term strategic orientation, commitment to intra-organizational fairness and diversity, and encouragement of knowledge sharing and critical thinking (Piwowar-Sulej & Iqbal, 2023). Moreover, they actively promote employee participation in decision-making, demonstrate concern for employees’ personal and professional development, and consistently provide support and guidance to facilitate employee growth (Avery & Bergsteiner, 2011). These practices foster a work environment conducive to psychological safety, trust, and empowerment, thereby enhancing employees’ engagement in organizational life and facilitating behaviors aligned with long-term sustainability objectives (Iqbal et al., 2020).

Drawing on the COR theory (Hobfoll et al., 2018), we propose that sustainable leadership fosters taking charge behavior—defined as discretionary, constructive efforts to change work processes or improve organizational functioning—in two critical ways.

First, sustainable leaders contribute to employees’ resource accumulation by offering task-related support, development opportunities, and emotional backing. According to the COR theory, individuals who possess abundant resources are more inclined to engage in proactive behaviors, as they have both the capacity and motivation to invest resources to obtain future gains (Hobfoll et al., 2018). Employees who benefit from resource-rich environments are likely to adopt a resource investment strategy, wherein they reciprocate leaders’ support by initiating constructive change efforts that signal loyalty, competence, and initiative (Halbesleben & Wheeler, 2015). For example, Pellegrini et al. (2018) found that when leaders actively support sustainability initiatives and provide resource-based incentives, employees demonstrate greater proactive engagement and responsiveness to organizational challenges.

Second, organizational justice, a cornerstone of sustainable leadership, strengthens employees’ perceptions that their contributions will be acknowledged and fairly rewarded. This sense of fairness enhances employees’ intrinsic motivation and self-efficacy, which are essential for engaging in high-risk behaviors like taking charge. Empirical research substantiates this link: Moon et al. (2008) demonstrated that both procedural and distributive fairness significantly predict proactive employee behavior. Similarly, Cantor et al. (2012) observed that employees are more inclined to initiate green change initiatives when they perceive organizational recognition and reward systems that align with sustainability values.

Collectively, these insights suggest that when employees perceive their organization as supportive, fair, and future-focused—key attributes of sustainable leadership—they are more willing and able to engage in taking charge behavior. This proactive behavior, in turn, contributes to organizational adaptability, innovation, and sustainable transformation. Accordingly, we hypothesize:

**H1.**  *Sustainable leadership is positively related to taking charge behavior*.

### 2.2. The Mediating Role of Employee Resilience

Building on the COR theory, we contend that employee resilience serves as a crucial mediating mechanism linking sustainable leadership to taking charge behavior. The COR theory posits that individuals are inherently motivated to acquire, preserve, and invest valuable resources to secure their future well-being and mitigate potential threats (Hobfoll et al., 2018). This dynamic process of resource management involves an initial phase of accumulation, followed by the strategic investment of amassed reserves. Within this theoretical framework, employee resilience emerges as a pivotal internal resource. We argue that resilience is not merely a consequence of resource acquisition but actively facilitates the strategic deployment and utilization of resources, thereby empowering employees to engage in proactive behaviors such as taking charge.

#### 2.2.1. Sustainable Leadership and Employee Resilience

Resilience is conceptualized as a personal psychological resource that enables individuals to effectively cope with adversity, setbacks, and evolving opportunities (Avey et al., 2009). In organizational settings, resilience is not a static trait but rather an evolving capacity, shaped significantly by contextual factors—particularly leadership (Cooke et al., 2019). Sustainable leadership plays a dual role in cultivating employee resilience, functioning both as a protective buffer and a proactive enabler. First, sustainable leaders emphasize employee development and adopt non-punitive management practices, thereby fostering a psychologically safe and secure work environment (Piwowar-Sulej & Iqbal, 2023). For instance, during transitions to green production, such leadership reduces employees’ perceived risks regarding career uncertainty, which helps preserve and reinforce their resilience (Richard, 2020).

Second, sustainable leaders actively enhance resilience by offering instrumental support, training, and growth opportunities—both for task performance and broader personal development (Piwowar-Sulej & Iqbal, 2023; Avery & Bergsteiner, 2011). This support equips employees with the skills and resources necessary to manage the uncertainties and challenges posed by green production initiatives. As S. Chen et al. (2015) note, employees who have access to diverse resources and competencies are better positioned to adapt to change and achieve their goals, thereby strengthening their resilience.

Thus, we propose the following hypothesis:

**H2.**  *Sustainable leadership is positively related to employee resilience*.

#### 2.2.2. Employee Resilience and Taking Charge

We propose that employee resilience fosters taking charge behavior through two interrelated psychological mechanisms: by amplifying intrinsic motivation and mitigating perceived risk.

First, resilience functions as a core psychological resource that activates employees’ intrinsic drive to engage in discretionary and future-oriented behaviors (Hobfoll et al., 2015, 2018). Resilient individuals are more inclined to transcend formal job expectations and immerse themselves in their tasks with greater initiative and persistence, motivated by aspirations for recognition, demonstration of competence, and long-term career progression (Xu et al., 2023). Empirical support for this perspective is found in the green hospitality sector, where Arasli et al. (2020) observed that employees with higher levels of psychological resilience were significantly more likely to take charge by initiating environmentally responsible practices that exceeded their formal role requirements.

Second, resilience serves as a psychological buffer that attenuates the inhibitory effects of fear and uncertainty commonly associated with high-risk behaviors such as taking charge. Whereas the anticipation of failure, resistance, or negative evaluation may discourage proactive action, resilient employees are more likely to appraise their environment as supportive and manageable, thus enhancing their perceived control and self-efficacy in the face of challenge (Newman et al., 2017). In contexts characterized by strategic transformation—such as the implementation of green production—this resilience enables employees to confront procedural ambiguity and technological change with greater confidence and adaptive capability (McAllister et al., 2007; Parker et al., 2010). Further evidence from Afshar Jahanshahi et al. (2021) in the service industry suggests that resilient employees exhibit lower risk aversion and are more likely to proactively engage in improving work processes, thereby aligning individual agency with organizational sustainability objectives.

Taken together, these insights suggest that resilience not only equips employees with the psychological readiness to act but also reduces the perceived costs of doing so, thereby creating a favorable motivational and cognitive foundation for taking charge. Accordingly, we propose the following hypothesis:

**H3.**  *Employee resilience is positively related to taking charge behavior*.

Overall, sustainable leaders, through consistent support for skill development, providing opportunities for voice, and ensuring fair treatment, not only protect employees from resource depletion but also foster the cultivation of adaptive capacities, with psychological resilience emerging as paramount (Piwowar-Sulej & Iqbal, 2023; Iqbal et al., 2020). Such resilient employees, characterized by enhanced adaptability, stress tolerance, and goal-directed motivation, are particularly well-equipped to navigate organizational challenges effectively. Consequently, we argue that sustainable leadership indirectly promotes taking charge behavior by cultivating employee resilience, which, in turn, provides the essential psychological and motivational foundation for initiating constructive organizational change. Building on this integrated theoretical rationale and the preceding hypotheses (H1–H3), we propose the following mediating hypothesis:

**H4.**  *Employee resilience acts as a mediator in the relationship between sustainable leadership and employees’ taking charge behaviors*.

### 2.3. Colleague Support as a Moderator

According to the Resource Caravan Passageways principle of the COR theory, individuals gradually accumulate resources, a process heavily influenced by environmental dynamics that either facilitate or impede resource acquisition (S. Chen et al., 2015). Within organizational contexts, colleague support constitutes a critical environmental factor (Bullough et al., 2014), significantly influencing how employees are treated and regarded in the workplace (Eisenkraft et al., 2017). This context profoundly shapes employees’ perceptions of resource availability and their psychological readiness to actively seek limited organizational resources.

Colleague support is typically categorized into two dimensions: emotional support, characterized by encouragement, recognition, and empathy, and instrumental support, characterized by knowledge sharing and practical assistance with work tasks (Halbesleben & Wheeler, 2015; Cohen & Wills, 1985). We argue that each of these support dimensions uniquely and meaningfully influences the process through which employees seek and acquire resources provided by sustainable leaders. Firstly, emotional support from colleagues fosters a psychologically safe workplace atmosphere (Tse et al., 2017). This atmosphere enables employees to proactively pursue valuable resources such as favorable organizational policies, job security, and developmental assistance from sustainable leaders without concerns about negative peer reactions or workplace repercussions. Consequently, emotional support enhances employees’ confidence and motivation in seeking leaders’ assistance to cultivate personal and internal resources. Secondly, instrumental support from colleagues mitigates the tendency toward excessive intra-group competition over limited resources (Halbesleben & Wheeler, 2015). By promoting cooperation and knowledge sharing, instrumental support allows employees to more readily and fully benefit from tools, task-related assistance, and other tangible resources provided by sustainable leaders. Conversely, in contexts lacking sufficient colleague support, employees may feel reluctant to actively seek resources from leaders due to concerns about jeopardizing collegial relationships. Additionally, heightened peer competition in such contexts can significantly restrict employees’ access to resources offered by sustainable leaders (Xanthopoulou et al., 2008). Based on the above rationale, we propose the following hypothesis:

**H5.**  *Colleague support positively moderates the relationship between sustainable leadership and employee resilience, with this effect stronger at higher levels of colleague support*.

**Figure 1:**
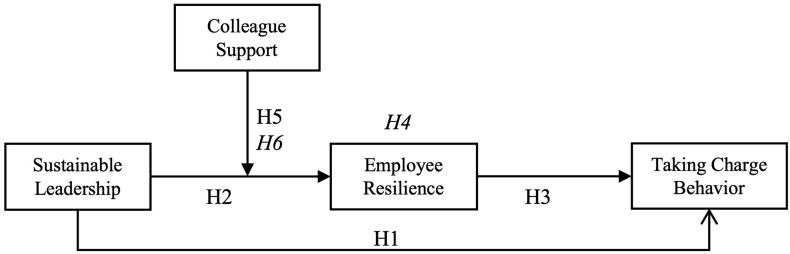
Theoretical framework of this study. (Notes: *H4*—Mediation Effect, *H6*—Moderated Mediation Effect).

Up to this point, we have discussed how sustainable leadership may impact taking charge behavior by affecting employee resilience and proposed the moderating role of colleague support in the link between sustainable leadership and taking charge behavior. Building on this, we posit that colleague support will influence the indirect relationship between sustainable leadership and taking charge behavior through employee resilience (see Figure 1). This influence stems from the notion that sustainable leadership is more likely to enhance employee resilience when colleague support levels are higher. Consequently, we present the following hypothesis:

**H6.**  *Colleague support positively moderates the mediating effect of employee resilience between sustainable leadership and employees’ taking charge behaviors. Specifically, this positive relationship is stronger at higher levels of colleague support*.

## 3. Research Methodology

### 3.1. Sampling and Data Collection

Data collection commenced in December 2024 via Credamo, a Chinese online survey platform with a large pool of registered users from diverse industries. To reduce sampling bias, we employed a randomized distribution approach within the platform’s user pool, inviting employees from a variety of industries. This approach, widely adopted in similar organizational research, supports efficient and reliable data collection within a defined period (Dey et al., 2022). Consistent with our focus on taking charge behavior in green production contexts, the study targeted industries known for substantial implementation of green production practices, including electronics, automotive, food, home appliances, and textile manufacturing. Firms within these sectors commonly adopt green initiatives—for example, textile companies employing eco-friendly dyes and advanced wastewater treatment systems, and electronics manufacturers upgrading production lines to reduce energy consumption.

To minimize measurement bias, we adopted a two-phase, leader-subordinate paired data collection approach, consistent with recommendations from prior research (Podsakoff et al., 2012). A two-week interval, as demonstrated by Ren et al. (2024), helps mitigate single-source bias. Prior to participation, all respondents were assured of anonymity, data confidentiality, and exclusive use of data for academic research.

In the initial phase (T1), employees completed surveys on sustainable leadership, perceived colleague support, and demographics. Two weeks later (T2), employees reported their resilience and control variables (such as green transformational leadership and individual environmental values), while supervisors assessed employee taking charge behavior and control variables (including organizational green climate and environmental regulatory pressures). To ensure data coherence across phases, user IDs and usernames were collected via the online system. Participants received a small incentive for their participation.

Before data collection, a power analysis was conducted using G*Power 3.1 following the guidelines of Faul et al. (2007). Assuming a moderate effect size (f^2^ = 0.15) and α = 0.05, the analysis indicated a minimum sample size of 184. To ensure adequate statistical power and robustness, 500 questionnaires were administered at T1, yielding 472 valid responses after excluding 28 inattentive responses. At T2, 472 questionnaires were distributed, resulting in 401 matched leader–subordinate pairs (85% response rate). After removing inattentive responses, 386 paired samples were retained for final analysis.

The final sample comprised participants from the electronics (*n* = 53, 13.7%), automotive (*n* = 77, 19.9%), food (*n* = 48, 12.4%), home appliances (*n* = 37, 9.6%), textile manufacturing (*n* = 52, 13.5%), and other manufacturing sectors (*n* = 119, 30.8%), which is broadly representative of current workforce distributions in these industries. Regarding demographics, 51.6% were male, and 76.7% were staff-level employees. Participants’ average age was 30.36 years (SD = 7.49). In terms of education, 18.4% held a master’s degree or higher, 76.4% held a bachelor’s degree, and 6.2% had a high school education or lower.

### 3.2. The Measurement of the Constructs

In this study, all measurement tools employed were validated scales from previous research, which were then translated into Chinese versions using the translation-back translation method (Reynolds et al., 1993). Participants rated all items on a 5-point Likert scale, with responses varying from 1 (strongly disagree) to 5 (strongly agree).

Sustainable Leadership. We used the Sustainable Leadership Questionnaire (SLQ) developed by McCann and Sweet (2013) to measure sustainable leadership. The SLQ is a well-established and validated scale in sustainable leadership research, with demonstrated applicability in the Chinese context (Xin et al., 2024). This questionnaire comprises 15 items, renowned for its strong reliability and validity (e.g., Iqbal et al., 2020), with sample statements such as “My leader has a plan to demonstrate sustainability when hiring, promoting employees, and replacing leaders.” Confirmatory factor analysis (CFA) affirmed the unidimensional structure of the SLQ within our sample (χ^2^ = 265.13, df = 90, CFI = 0.94, TLI = 0.93, RMSEA = 0.071). All items loaded significantly onto the latent factor, with standardized loadings ranging from 0.63 to 0.76. This structural pattern aligns with the original factor configuration reported in the English version of the SLQ. Furthermore, the scale demonstrated excellent internal consistency, with a Cronbach’s alpha of 0.94.

Employee Resilience. It was evaluated using the Employee Resilience Scale (ERS) devised by Näswall et al. (2019). This scale is self-rated by employees and places specific emphasis on the workplace context, as opposed to general measures of individual resilience, consisting of 9 items, with sample statements include “I successfully manage a high workload for long periods of time.” (Cronbach’s α = 0.90).

Colleague Support. To evaluate this, we utilized the Perceived Colleague/Peer Support (PCS) scale, adapted by Simosi (2012) from Eisenberger et al. (1986). This scale, assessed by employees based on their perceptions, comprises 6 items, with a sample item being “Help is available from colleagues when I have a problem.” (Cronbach’s α = 0.87).

Taking Charge Behavior. We evaluated it using the tool developed by Morrison and Phelps (1999). The scale consists of 10 items, with a representative statement being “I try to implement solutions to pressing organizational problems.” (Cronbach’s α = 0.91).

### 3.3. Control Variables

Drawing from previous research on sustainable leadership, employee resilience, and taking charge behaviors, and guided by the COR theory, we identified and incorporated several pertinent control variables into our analytical framework. Consistent with established empirical practices (Dey et al., 2022), demographic factors including respondents’ gender, age, education level, and hierarchical rank were collected and controlled, given their demonstrated influence on employee resilience and proactive behaviors within organizational contexts (Newman et al., 2017; Parker et al., 2010).

Moreover, to account for potentially confounding effects beyond demographic characteristics, we included additional theoretically relevant organizational and individual-level variables. Specifically, consistent with prior literature examining employee environmental behaviors (Chou, 2014; Islam et al., 2021), we controlled for organizational green climate, individual environmental values, and perceived environmental regulatory pressures. These contextual and individual difference variables are critical in shaping employees’ motivational states and capabilities to proactively engage in taking charge behaviors within green production scenarios.

Finally, given the previously documented relationship between green transformational leadership and employee taking charge behaviors (Du & Yan, 2022), we also included green transformational leadership as an essential control variable. By doing so, we effectively isolate the distinct contribution of sustainable leadership to employees’ taking charge behaviors from that attributable to environmentally focused transformational leadership, thereby clarifying the unique effects of sustainable leadership within our conceptual model.

For control variables beyond demographic information, we used scales that were validated in the Chinese context. All items were rated on a 5-point Likert scale, with responses ranging from 1 (strongly disagree) to 5 (strongly agree). Specifically, we used the scale developed by Y. S. Chen and Chang (2013) to measure green transformational leadership (Cronbach’s α = 0.91), consisting of 6 items, with a representative statement such as “My leader provides a clear environmental vision for the project members to follow.” We used Steg et al.’s (2005) tool to measure individual green values (Cronbach’s α = 0.87), which includes 6 items, with a sample item being “I feel normally obliged to protect the environment instead of causing degradation.” We used Dumont et al.’s (2017) tool to measure the green organizational climate (Cronbach’s α = 0.90), consisting of 6 items, with a sample item such as “Our company cares about how to become more environmentally friendly.” Finally, we used Cai and Li’s (2018) tool to measure the company’s environmental regulation pressures (Cronbach’s α = 0.88), which includes 3 items, with a representative item being “Our products need to comply with national environmental regulations.”

## 4. Results

### 4.1. Analysis and Findings

We utilized SPSS 26 and M plus 8 for data analysis. Prior to hypothesis testing, we evaluated the convergent and discriminant validity of the measurement tools used in this study through confirmatory factor analysis (CFA). All scales exhibited strong convergent validity, with factor loadings exceeding 0.70, the average variance extracted (AVE) for each scale exceeding 0.50, and the composite reliability (CR) surpassing 0.70 (refer to Table 1). To assess discriminant validity, two approaches were employed. Firstly, we compared various factor models (refer to Table 2). An initial model with sustainable leadership, employee resilience, colleague support, and taking charge behavior was specified, showcasing a good fit. The four-factor model outperformed a three-factor model that combined sustainable leadership and colleague support into one factor, as well as a two-factor model merging the variables other than the dependent variables. Secondly, the square root of each AVE value exceeded the correlations among factors, according to Fornell and Larcker (1981) (refer to Table 3). These results validate the strong discriminant validity among the study variables.

Given the reliance on questionnaire data and potential for common source bias, we examined for common method variance (CMV). This analysis included Harman’s single-factor test on the four core variables, which uncovered four factors with eigenvalues exceeding 1. The first component explained for 30.57% of the variance, notably below the 40% threshold, as highlighted by Podsakoff et al. (2012). Additionally, the CFA demonstrated that the single-factor model inadequately fit the data (χ^2^/df = 6.132; GFI = 0.447; RMSEA = 0.115; RMR = 0.093; CFI = 0.535). Consequently, there is no significant evidence of common method variance present in this study.

Table 3 presents the means, standard deviations, and correlation coefficients for the key variables in this study. The results indicate a significant positive correlation between sustainable leadership and both employee resilience (r = 0.42, *p* < 0.01) and taking charge behavior (r = 0.40, *p* < 0.01). Additionally, a significant positive correlation is observed between employee resilience and taking charge behavior (r = 0.42, *p* < 0.01), providing preliminary evidence for the upcoming hypotheses testing.

### 4.2. Hypothesis Tests

Hierarchical regression analysis was employed to test the hypotheses. Hypothesis 1 posited that sustainable leadership would have a positive impact on taking charge behavior. In Model 5 of Table 4, sustainable leadership exhibit a positive association with taking charge behavior (β = 0.35, *p* < 0.01), even after accounting for variables such as gender and age. Therefore, Hypothesis 1 received empirical support.

Hypotheses 2, 3, and 4 proposed that sustainable leadership would positively impact employee resilience, subsequently influencing taking charge behavior, with employee resilience acting as a mediator between sustainable leadership and taking charge behavior. Model 2 in Table 4 reveals a significant positive correlation between sustainable leadership and employee resilience (β = 0.36, *p* < 0.01), while Model 6 demonstrates a significant positive correlation between employee resilience and taking charge behavior (β = 0.28, *p* < 0.01). Using the PROCESS macro plugin in SPSS (Hayes, 2013), a path analysis with bootstrapping (bootstrap sample size = 20,000) was conducted to further explore the mediating effects, as recommended by Edwards and Lambert (2007). The findings indicate an indirect effect of sustainable leadership on taking charge behavior, with a 95% CI of [0.05, 0.14], providing empirical support for Hypotheses 2, 3, and 4.

**Figure 2:**
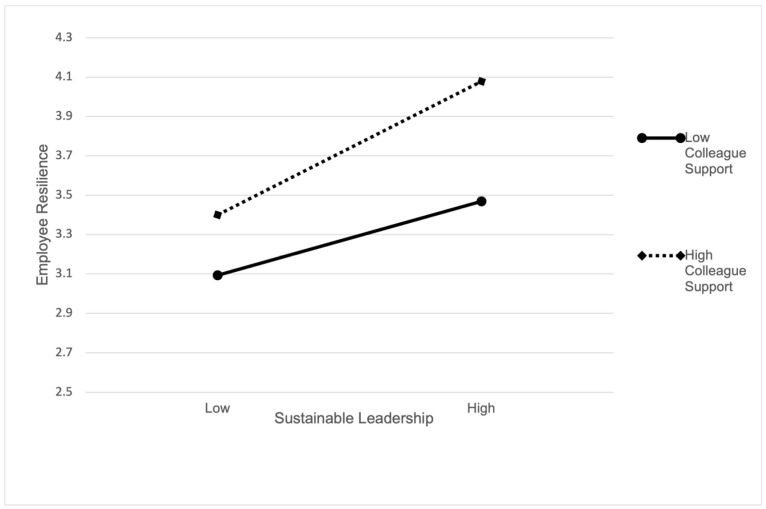
Moderating effect of colleague support.

Hypothesis 5 proposed that colleague support acts as a positive moderator in the relationship between sustainable leadership and employee resilience, indicating that the influence of sustainable leadership on employee resilience strengthens with higher colleague support levels and diminishes otherwise. Model 3 in Table 4 demonstrates a significant positive correlation between the interaction term of sustainable leadership and colleague support with employee resilience (β = 0.18 *p* < 0.05), confirming a substantial moderating effect of colleague support. To deepen comprehension of this moderating impact, we graphed the moderation effect of sustainable leadership on employee resilience at high (+1 SD) and low (−1 SD) levels of colleague support (depicted in Figure 2). At higher levels of colleague support, sustainable leadership significantly correlates with employee resilience (β = 0.36, *p* < 0.01, 95% CI = [0.25, 0.47]), whereas at lower levels of colleague support, the association remains significant (β = 0.18, *p* < 0.01, 95% CI = [0.09, 0.26]), albeit with reduced strength. Consequently, Hypothesis 5 is corroborated.

Following the guidance of Edwards and Lambert (2007), we conducted tests on moderated mediation. Utilizing the PROCESS macro plugin in SPSS, we employed 20,000 bootstrapped samples to calculate bias-corrected confidence intervals for significance testing. As demonstrated in Table 5, at high level of colleague support (+1 SD), sustainable leadership exhibits a positive and significant indirect impact on taking charge behavior through employee resilience (β = 0.12, *p* < 0.01, 95% CI = [0.06, 0.20]). Conversely, at low level of colleague support (−1 SD), sustainable leadership still shows a positive and significant indirect effect on taking charge behavior through employee resilience (β = 0.05, *p* < 0.05, 95% CI = [0.01, 0.09]), albeit with a reduced impact. Hypothesis 6 is substantiated.

## 5. Discussion and Conclusions

In recent years, scholars have increasingly recognized the vital role of sustainable leadership in promoting both organizational and societal sustainability. As a result, there has been a shift in focus towards understanding how sustainable leadership influences employee behavior to facilitate these goals. This study specifically examines how sustainable leadership influences employee behavior, with a particular focus on taking charge behavior, to advance green production and sustainable development. Drawing on the COR theory, this research demonstrates that sustainable leadership enhances taking charge behavior by fostering employee resilience, particularly in supportive environments.

### 5.1. Results Analysis

This study presents several key findings.

Firstly, the findings of this study highlight the positive impact of sustainable leadership on employees’ taking charge behavior, supporting the notion that sustainable leadership encourages individuals to challenge established organizational norms (Piwowar-Sulej & Iqbal, 2023). This result suggests that when leaders provide support and foster a fair organizational environment, employees are more intrinsically motivated to proactively engage in the organization’s green production initiatives.

Secondly, our results offer empirical evidence for the mediating role of employee resilience in the relationship between sustainable leadership and taking charge behavior. This finding aligns with the work of Iqbal and Piwowar-Sulej (2022), which showed that sustainable leadership enhances employee resilience, and Caniëls and Baaten (2019), who found that resilience fosters proactive work behaviors. Specifically, our results indicate that sustainable leadership, through providing support and prioritizing employees’ needs, enhances their capacity for sustained career development, which in turn motivates them to actively contribute to the organization’s proactive initiatives.

Finally, this study confirms the hypothesis regarding the moderating role of colleague support in enhancing the effectiveness of sustainable leadership. Our finding is consistent with Z. Wang et al. (2024), who observed that coworker support influences both the transmission of knowledge-based resources from leaders and employees’ ability to absorb and transform these resources. This result suggests that in a supportive colleague environment, employees feel free to utilize available organizational resources and tools, while also being empowered to showcase their own contributions.

### 5.2. Theoretical Implications

This study contributes substantially to the existing literature on sustainable leadership and green production in three critical ways.

First, by examining employee taking charge behavior, this research extends the behavioral boundary of sustainable leadership. Prior studies predominantly focused on how sustainable leadership influences relatively low-risk behaviors, such as role-specific or team relationship-oriented behaviors. While insightful, these studies have not adequately explored sustainable leadership’s impact on riskier, proactive employee behaviors—such as taking charge—that are crucial for initiating sustained organizational change and generating long-term benefits (Li & Zhang, 2023; Moon et al., 2008). By positioning taking charge as a central outcome, this study highlights the expanded role sustainable leadership can play in stimulating bold, constructive initiatives essential to the long-term sustainability of organizations. This shift enriches the conceptualization of sustainable leadership’s role in driving transformative, sustainability-oriented organizational change.

Second, this research sheds new light on the internal mechanisms through which sustainable leadership influences proactive yet inherently risky employee behaviors. Existing theoretical frameworks, predominantly drawing upon Social Learning Theory and Social Exchange Theory, largely emphasize external cues and reciprocal exchanges, thus inadequately addressing the internal motivational processes underpinning risky proactive behaviors. By integrating the COR theory, this study provides a more detailed and nuanced explanation of how sustainable leadership functions—specifically, by helping employees accumulate psychological resources that bolster intrinsic motivation. By introducing employee resilience as a mediating factor, we explicate that sustainable leadership promotes proactive behaviors by enhancing internal psychological resources, which in turn stimulate intrinsic motivation and equip employees with the psychological capacities necessary to navigate risks effectively. This advancement provides a dynamic and multifaceted perspective on the mechanisms of sustainable leadership and extends the application of the COR theory into the domain of sustainable leadership and proactive organizational behavior.

Third, this study identifies colleague support as a critical boundary condition that shapes the effectiveness of sustainable leadership in promoting proactive behaviors. This responds directly to recent scholarly calls for additional examination of contextual moderators of sustainable leadership effectiveness (Iqbal et al., 2020). Positive colleague interactions substantially amplify sustainable leadership’s effectiveness by bolstering employees’ confidence in resource acquisition and minimizing detrimental intra-organizational competition over limited resources. This highlights the pivotal role of social context in shaping the effectiveness of sustainable leadership, particularly within the uncertainty-laden context of green production environments. By explicitly modeling this moderated mediation mechanism, this research advances theoretical insights into the interactive dynamics linking leadership practices, peer relationships, and employee proactive behavior, thereby broadening both the theoretical boundaries of sustainable leadership mechanisms and the practical applicability of the COR theory.

### 5.3. Practical and Policy Implications

The present findings yield several important managerial implications for promoting employees’ taking charge behavior within the context of green production.

First, the results highlight the critical role of sustainable leadership in fostering a proactive workforce capable of embracing and advancing organizational change. The significant influence of sustainable leadership necessitates developing specific competencies beyond general management skills. Organizations should implement targeted leadership development initiatives—such as structured training programs, action learning projects, and behavioral simulations—to cultivate sustainable leadership principles and practices (McCray et al., 2021). Enhanced sustainable leadership empowers employees by effectively facilitating resource access, thereby enabling discretionary change-oriented behaviors critical for advancing green production objectives.

Second, enhancing employee resilience can significantly magnify the influence of sustainable leadership on taking charge behavior. As organizations transition to green production, beyond the provision of external resources, organizations should invest in developing employees’ psychological resources through formal resilience-building programs. Formal resilience-building programs focusing on psychological capital (PsyCap) components—optimism, self-efficacy, and hope (Avey et al., 2009)—are essential. Such development bolsters adaptive capacity and confidence in managing the social and performance risks inherent in taking-charge initiatives, thereby increasing proactive engagement in organizational transformation.

Third, fostering a culture of mutual support among employees is essential for maximizing the behavioral impact of sustainable leadership, particularly within cultural contexts emphasizing relational interdependence (e.g., China’s guanxi system). Guanxi, characterized by reciprocal trust and obligation (X. P. Chen & Peng, 2008), fundamentally underpins psychological safety and peer support (Liu & Wang, 2013). Strong guanxi networks mitigate perceived relational risks when pursuing resources, facilitating taking charge. Managers should therefore actively promote relational embeddedness alongside task coordination. Implementing practices that enhance team cohesion, reduce counterproductive competition, and resolve conflict constructively (Tse et al., 2017) strengthens both formal and informal support structures. This creates a resource-rich environment essential for sustainable leadership efficacy in green production settings.

Collectively, these managerial strategies contribute to the development of a psychologically empowered, socially supported, and resource-abundant workforce—conditions under which employees are most likely to take charge and drive the organization’s green production transformation agendas.

### 5.4. Limitations and Future Research

This study has several limitations that offer avenues for future research. First, from a resource investment perspective, while this study highlights the unique characteristics of sustainable leadership and its dynamic effect on employees’ taking charge behavior, it does not fully capture the full range of mediating pathways through which this influence occurs. Employees may engage in taking charge behaviors due to factors beyond the scope of this study, such as skill development opportunities or extrinsic incentive systems. For example, Parker and Collins (2010) found that implementing an incentive system for taking charge can effectively stimulate employees’ motivation. Therefore, we encourage future research to explore additional mechanisms through which sustainable leadership promotes taking charge behavior.

Second, this study focused on employees within green production industries enhances internal validity but limits generalizability (Sun & Xi, 2024). The salience of environmental sustainability in these sectors likely amplifies the prevalence and impact of sustainable leadership. In industries where environmental concerns are less central (e.g., education, banking), sustainable leadership may be less common or exert weaker effects on proactive behaviors like taking charge. Hence, Further studies are encouraged to test the generalizability of our findings across different organizational and industrial environments.

## Figures and Tables

**Table 1:** Convergent Validity: AVE and CR for Each Scale.

Scales	AVE	CR
Sustainable Leadership Questionnaire	0.509	0.939
Perceived Colleague/Peer Support scale	0.575	0.89
Employee Resilience Scale	0.508	0.903
Taking Charge Behavior Scale	0.508	0.912
Green Transformational Leadership Scale	0.617	0.906
Individual Green Values Scale	0.572	0.888
Green Organizational Climate	0.601	0.9
Environmental Regulatory Pressures Scale	0.709	0.879

Notes: AVE = Average Variance Extracted; CR = Composite Reliability.

**Table 2:** Confirmatory factor analysis.

Factor Structure	χ^2^	df	χ^2^/df	GFI	RMSEA	RMR	CFI	TLI	NNFI
One-factor model (combining all items into one factor)	4537.73	740	6.132	0.447	0.115	0.093	0.535	0.510	0.510
Two-factor model (combining SL, ER, CS together)	3275.52	739	4.432	0.565	0.094	0.074	0.689	0.672	0.672
Three-factor model (combining SL and CS together)	2146.58	737	2.91	0.735	0.070	0.062	0.827	0.817	0.817
Four-factor model	1279.49	734	1.74	0.907	0.044	0.036	0.933	0.929	0.929

Notes: GFI = Goodness-of-fit index, RMSEA = Root-mean-square error of approximation, RMR = Root mean residua, CFI = Comparative fit index, TLI = Tucker–Lewis index, NNFI = Non-normed fit index. SL = Sustainable leadership, ER = Employee resilience, CS = Colleague support.

**Table 3:** Means, standard deviations, and correlation.

Variable	Mean	SD	1	2	3	4	5	6	7	8	9	10	11	12
Gender	—	—												
Age	30.36	7.49	−0.05											
Edu	—	—	0.06	−0.07										
Rank	—	—	0.02	0.49 **	0.09									
ERP	3.78	0.93	0.01	−0.08	−0.02	−0.08	(0.842)							
GTL	3.40	0.84	−0.05	0.02	−0.11	0.02	0.24 **	(0.786)						
GOC	3.44	0.88	0.00	0.08	−0.07	−0.01	0.26 **	0.33 **	(0.775)					
IGV	3.60	0.81	0.02	0.02	0.02	0.03	0.28 **	0.22 **	0.23 **	(0.757)				
SL	3.43	0.71	0.08	0.09	0.05	0.08	0.05	0.22 **	0.18 **	0.22 **	(0.713)			
CS	3.58	0.73	0.03	0.08	−0.06	0.08	0.12 *	0.10	0.13 **	0.02	0.25 **	(0.758)		
ER	3.80	0.56	0.07	0.07	0.03	0.17 *	0.05	0.23 **	0.15 **	0.19 **	0.42 **	0.33 **	(0.712)	
TCB	3.45	0.63	0.04	0.11 *	−0.08	0.12 *	0.09	0.21 **	0.24 **	0.19 **	0.40 **	0.17 **	0.42 **	(0.712)

Notes: Edu = Education, ERP = Environmental regulations pressures, GTL = Green transformation leadership, GOC = Green organizational climate, IGV = Individual green value, SL = Sustainable leadership, ER = Employee resilience, CS = Colleague support, TCB = Taking charge behavior. The values in parentheses represent the square root of each Average Variance Extracted (AVE) value. * *p* < 0.05, ** *p* < 0.01.

**Table 4:** Hierarchical regression analysis.

	Employee Resilience			Taking Charge Behavior		
	M1	M2	M3	M4	M5	M6
CV						
Gender	0.07 (0.05)	0.04 (0.05)	0.03 (0.05)	−0.03 (0.06)	−0.06 (0.06)	−0.07 (0.06)
Age	−0.02 (0.01)	−0.05 (0.01)	−0.06 (0.01)	0.03 (0.01)	0.01 (0.01)	0.02 (0.01)
Education	0.04 (0.06)	0.01 (0.06)	0.03 (0.05)	−0.06 (0.07)	−0.09 (0.06)	−0.09 (0.06)
Rank	0.17 ** (0.04)	0.16 ** (0.04)	0.15 (0.07)	0.11 (0.04)	0.10 (0.04)	0.06 (0.04)
ERP	0.04 (0.03)	0.02 (0.03)	0.02 (0.03)	0.01 (0.04)	0.02 (0.03)	0.03 (0.03)
GOC	0.07 (0.03)	0.03 (0.03)	−0.01 (0.03)	0.15 ** (0.04)	0.12 * (0.04)	0.11 (0.04)
LGV	0.13 ** (0.04)	0.07 (0.03)	0.07 (0.03)	0.13 * (0.04)	0.07 (0.04)	0.05 (0.04)
GTL	0.20 ** (0.04)	0.13 ** (0.03)	0.10 * (0.03)	0.13 * (0.04)	0.07 (0.04)	0.03 (0.04)
IV						
SL		0.36 ** (0.04)	0.34 ** (0.04)		0.35 ** (0.04)	0.25 ** (0.04)
Mediator						
ER						0.28 ** (0.06)
Moderator						
CS			0.25 **(0.04)			
Interaction						
SL × CS			0.14 *(0.02)			
R^2^	0.09	0.21	0.28	0.09	0.20	0.26
∆R^2^	0.09	0.12	0.07	0.09	0.11	0.06
∆F	5.92 **	57.61 **	8.80 **	5.83 **	51.64 **	31.45 **

Notes: CV = Control variables, IV = Independent variables, ERP = Environmental regulations pressures, GTL = Green transformation leadership, GOC = Green organizational climate, LGV = Individual green value, SL = Sustainable leadership, ER = Employee resilience, CS = Colleague support. * *p* < 0.05; ** *p* < 0.01.

**Table 5:** Moderated mediating effect test.

Moderator	β	SE	LLCI	ULCI
Low	0.05	0.02	0.02	0.10
Mean	0.09	0.02	0.05	0.14
High	0.12	0.04	0.06	0.19

Notes: LLCI = Lower level confidence interval; ULCI = Upper level confidence interval.

## Data Availability

We have arranged with the participants in our study to keep our data private but will provide it upon reasonable request. Therefore, the datasets generated and/or analyzed during the current study are available from the corresponding author upon reasonable request.
